# Malaria and global warming in perspective?

**DOI:** 10.3201/eid0603.000315

**Published:** 2000

**Authors:** P Martens


and other gram-negative pathogens in Europe (8-10) underlines the need for systematic surveillance to monitor the spread of similar resistance determinants.

This work was supported by grants no. FMRX-CT98-0232 from the European TMR Research Network on metallo-ß-lactamases and no. 9906404271 from MURST ex-40%.

**Gian Maria Rossolini,* Maria Letizia Riccio,* Giuseppe Cornaglia,† Laura Pagani,‡ Cristina Lagatolla,§ Laura Selan,¶ and Roberta Fontana†**

* Università di Siena, Siena, Italy; †Università di Verona, Verona, Italy; ‡Università di Pavia, Pavia, Italy; §Università di Trieste, Trieste, Italy; and ¶Università "La Sapienza," Rome, Italy

### References

1. Livermore DM. Acquired carbapenemases. J Antimicrob Chemother 1997;39:673-6.
2. Rasmussen BA, Bush K. Carbapenem-hydrolyzing ß-lactamases. Antimicrob Agents Chemother 1997;41:223-32.
3. Lauretti L, Riccio ML, Mazzariol A, Cornaglia G, Amicosante G, Fontana R, et al. Cloning and characterization of ***bla***$_{VIM}$, a new integron-borne metallo-ß-lactamase gene from a ***Pseudomonas aeruginosa*** clinical isolate. Antimicrob Agents Chemother 1999;43:1584-90.
4. Mazzariol A, Cornaglia G, Piccoli P, Lauretti L, Riccio ML, Rossolini GM, et al. Carbapenem-hydrolyzing ß-lactamases in ***Pseudomonas aeruginosa***. Eur J Clin Microbiol Infect Dis 1999;18:455-6.
5. Senda K, Arakawa Y, Nakashima K, Ito H, Ichiyama S, Shimokata K, et al. Multifocal outbreaks of metallo-ß-lactamase-producing ***Pseudomonas aeruginosa*** resistant to broad-spectrum ß-lactams, including carbapenems. Antimicrob Agents Chemother 1996;40:349-53.
6. Lee K, Chong Y, Shin HB, Yong D. Rapid increase of imipenem-hydrolyzing ***Pseudomonas aeruginosa*** in a Korean hospital. In: Program and Abstracts of the 38th Interscience Conference on Antimicrobial Agents and Chemotherapy. Washington: American Society for Microbiology; 1998. [Abstract E85].
7. Koh TH, Babini GS, Woodford N, Sng LH, Hall LM, Livermore DM. Carbapenem-hydrolysing IMP-1 ß-lactamase in ***Klebsiella pneumoniae*** from Singapore. Lancet 1999;353:2162.
8. Cornaglia G, Riccio ML, Mazzariol A, Lauretti L, Fontana R, Rossolini GM. Appearance of IMP-1 metallo-ß-lactamase in Europe. Lancet 1999;353:899-900.
9. Woodford N, Palepou M-FI, Babini GS, Bates J, Livermore DM. Carbapenemase-producing ***Pseudomonas aeruginosa*** in the UK. Lancet 1998;352:546-7.
10. Cardoso O, Sousa JC, Leitâo R, Peixe L. Carbapenem-hydrolysing ß-lactamase from clinical isolates of ***Pseudomonas aeruginosa*** in Portugal. J Antimicrob Chemother 1999;44:135.

### Malaria and Global Warming in Perspective?

**To the Editor:** I read with great interest the article "From Shakespeare to Defoe: malaria in England in the Little Ice Age" (1). Unfortunately, the article is not as balanced as a presentation last year by Paul Reiter, which clearly illustrated that, although climate is important in the transmission of malaria, the influence of other factors (e.g., access to medical care and improved housing) is likely to be of more importance in Europe.

Malaria indeed was quite common in Europe, even in the Roman Empire and in Medieval Europe, and until a few decades ago, it was still present in parts of Europe, Australia, and North America. In fact, the failure of the 1806 British invasion of Zeeland in the Netherlands may be attributable to infection of the British forces with malaria. However, the authors referenced by Reiter have never made the claim that in the coming years warmer "temperatures will result in malaria transmission in Europe and North America." On the contrary, the reports of the Intergovernmental Panel on Climate Change Reiter quotes conclude that "Although climate change could increase the potential transmission of malaria [in Europe and North America], existing public health resources—disease surveillance, surface water management, and treatment of cases—would make reemergent malaria unlikely" (2,3).

Reiter's argument that some scientists attribute the recent observed increase in malaria risk to climate trends is also not accurate. While acknowledging the sensitivity of the malaria mosquito and parasite to climate, these researchers examine insect and incidence data to explore multiple factors underlying malaria emergence. Another group of scientists uses mathematical simulation models to estimate changes in malaria risk over the next few decades. These models, which are heuristic tools not meant to predict future worlds, assess how potential risk for malaria may by affected by changes in climate (4). The goals of both types of research are to improve knowledge of the complex malaria transmission cycle, define epidemic-prone areas, identify the reasons for increased malaria risk, and develop solutions to protect vulnerable communities.

Dr. Reiter acknowledges the sensitivity of malaria to climatic influences, and I am sure that he agrees that change in climate will affect risk for transmission—he may be skeptical as to whether global warming will ever become a fact, but that is another question. While Reiter's paper offers an interesting perspective on the history of malaria in Europe, it provides no illuminating information on the influence of climate change on human health.

**Pim Martens**
Maastricht University, Maastricht, the Netherlands

### References

1. Reiter P. From Shakespeare to Defoe: malaria in England in the Little Ice Age. Emerg Infect Dis 2000;6:1-11.
2. Intergovernmental Panel on Climate Change. The regional impacts of climate change: an assessment of vulnerability. Working Group II. Intergovernmental Panel on Climate Change. New York: Cambridge University Press; 1998. Chapters 5, 8.
3. Intergovernmental Panel on Climate Change. Climate change 1995: impacts, adaptations and mitigation of climate change: scientific-technical analyses. Working Group II, Intergovernmental Panel on Climate Change. New York: Cambridge University Press; 1996. Chapter 18.
4. Martens P. Health and climate change: modelling the impacts of global warming and ozone depletion. London: Earthscan Publications Ltd.; 1998.

For P. Reiter's response, please see
http://www.cdc.gov/ncidod/EID/vol6no4/reiter.htm

### Serologic Evidence of Human Monocytic and Granulocytic Ehrlichiosis in Israel

**To the Editor:** We read with great attention the article by Dr. Keysary et al., who reported the first evidence of human monocytic and granulocytic ehrlichiosis in Israel (1); however, we disagree with their conclusions.

Ehrlichiae comprise a large group of intracellular organisms pathogenic for animals and occasionally for humans. Because these organisms are closely related, serologic cross-reactions occur within and between groups, leading to mistakes in identification. For example, ***Ehrlichia chaffeensis*** was misdiagnosed as ***E. canis*** in humans (2) and human granulocytic ehrlichiosis as human monocytic ehrlichiosis in areas where the vector was not present (3). Because of such cross-reactions, serology alone is not sufficient to establish the existence of a new ehrlichial disease.

With the exception of ***Rhipicephalus sanguineus***, the brown dog tick, which is distributed worldwide, tick species of medical importance are very geographically specific. For example, the ***Ixodes*** and ***Dermacentor*** spp. found in Europe are not those found in the United States. Consequently, tick-transmitted organisms and diseases are also very specific geographically. For example, ***Borrelia*** spp. found in the Old World are not found in America (except for ***B. burgdorferi stricto sensu,*** which is found in both Europe and America). ***R. rickettsii***, transmitted by ***Dermacentor andersoni*** and ***D. variablilis,*** is reported in the United States but not in Europe, where the vectors are not present.

American monocytic ehrlichiosis is caused by ***E. chaffeensis***, which is transmitted by the tick ***Amblyomma americanum,*** found only in America. The main reservoir is the deer ***Odocoileus virginanus*** (4).

It is very unlikely that a tick-borne disease occurred in a country where neither the vector nor the reservoir of the bacterium exists. All attempts to demonstrate the presence of ***E. chaffeensis*** in the Old World, including Africa, have failed. Indeed, there is no convincing evidence of the existence of ***E. chaffeensis*** outside America.

**Philippe Brouqui* and J. Steven Dumler†**
Unité des Rickettsies, Faculté de Médecine, Marseille, France; and Johns Hopkins University School of Hygiene & Public Health, Baltimore, Maryland, USA

### References

1. Keysary A, Amram L, Keren G, Sthoeger Z, Potasman I, Jacob A, et al. Serologic evidence of human monocytic and granulocytic ehrlichiosis in Israel. Emerg Infect Dis 1999;5:775-8.
2. Maeda K, Markowitz N, Hawley RC, Ristic M, Cox D, McDade JE. Human infection with ***Ehrlichia canis***, a leukocytic rickettsia. N Engl J Med 1987;316:853-6.
3. Brouqui P, Raoult D. Human ehrlichiosis. N Engl J Med 1994;330:1760-1.
4. Dumler JS, Bakken JS. Ehrlichial diseases of humans: emerging tick-borne infections. Clin Infect Dis 1995;20:1102-10.

